# Possible Involvement of *MYB44*-Mediated Stomatal Regulation in Systemic Resistance Induced by *Penicillium simplicissimum* GP17-2 in *Arabidopsis*

**DOI:** 10.1264/jsme2.ME16025

**Published:** 2016-06-10

**Authors:** Ayaka Hieno, Hushna Ara Naznin, Mitsuro Hyakumachi, Mieko Higuchi-Takeuchi, Minami Matsui, Yoshiharu Y. Yamamoto

**Affiliations:** 1 The Graduate School of Agricultural Science, Gifu UniversityYanagido 1–1, Gifu 501–1193Japan; 2 Applied Biological Sciences, Gifu UniversityYanagido 1–1, Gifu 501–1193Japan; 3 RIKEN CSRSSuehiro-cho 1–7–22, Tsurumi-ku, Yokohama-shi, Kanagawa, 230–0045Japan; 4 JST ALCAJapan

**Keywords:** *MYB44*, plant growth-promoting fungi (PGPF), *Pseudomonas syringae* pv. *tomato* DC3000, stomatal resistance

## Abstract

The plant growth-promoting fungus (PGPF), *Penicillium simplicissimum* GP17-2 (GP17-2), induces systemic resistance against *Pseudomonas syringae* pv. *tomato* DC3000 (*Pst*) in *Arabidopsis thaliana*. The molecular mechanisms underlying induced systemic resistance (ISR) by GP17-2 were investigated in the present study. Microscopic observations revealed that stomatal reopening by *Pst* was restricted by elicitation with the culture filtrate (CF) from GP17-2. A gene expression analysis of *MYB44*, which enhances abscisic acid signaling and consequently closes stomata, revealed that the gene was activated by CF. CF-elicited *myb44* mutant plants failed to restrict stomatal reopening and showed lower resistance to *Pst* than wild-type plants. These results indicate that stomatal resistance by GP17-2 is mediated by the gene activation of *MYB44*. We herein revealed that the *MYB44*-mediated prevention of penetration through the stomata is one of the components responsible for GP17-2-elicited ISR.

Plants are surrounded by a large number of microorganisms, some of which improve plant adaptation to diverse stresses. Plant growth-promoting fungi (PGPF) are mutualistic microbes (24). We previously characterized the mutualistic activity of the PGPF isolate, *Penicillium simplicissimum* GP17-2 (GP17-2), which was isolated from the rhizosphere of zoysiagrass (*Zoysia tenuifolia*) (18, 19). The culture filtrate (CF) from GP17-2 has the ability to elicit induced systemic resistance (ISR) in plants, effectively controlling several diseases such as anthracnose (13, 27) and angular leaf spot in the cucumber (13), *cucumber mosaic virus* (CMV) in *Arabidopsis* and tobacco (4), and bacterial leaf speck in *Arabidopsis* (6).

Plant pathogenic bacteria invade plants through natural openings, such as stomata and water pores, or accidental wounds. Stomatal closure is an essential part of innate immunity against bacterial pathogens (2, 3, 14, 15, 20–22, 28, 31–33). In order to overcome stomatal closure by plants, *Pseudomonas syringae* pv. *tomato* DC3000 (*Pst*) forces stomatal reopening with the phytotoxin coronatine (COR) (34). Since the chemical structure of COR is similar to that of jasmonoyl-isoleucine (JA-Ile), COR has been suggested to activate JA signaling as an analog of JA-Ile in order to reopen stomata (20). A root treatment with abscisic acid (ABA) and salicylic acid (SA) was previously shown to restrict stomatal reopening by *Pst* (14), suggesting the involvement of ABA-and/or SA-mediated signaling in ISR.

We have shown that SA- and jasmonate (JA)/ethylene (ET)-responsive genes were both activated by a root treatment with CF (6). Single mutants unable to transduce SA, JA, or ET signals alone still retained the ability to induce CF-elicited ISR (6). Based on these findings, we hypothesized that ISR is transduced by multiple signaling pathways in parallel, including the SA, JA, and ET pathways (6). We also observed that some ABA responsive genes, namely *LEAs*, *RAB18*, *RD22*, *GOLS2*, and *ABI5*, were activated by CF (data not shown). These findings prompted us to investigate whether ABA signaling is also involved in ISR.

The transcription factor, *MYB44* (AT5G67300), has been identified as a stomata-specific enhancer of the ABA signal for stomatal closure (9). We were motivated to perform the study by the elicitor responsibility of *MYB44* (8, 16, 24). We herein focused on *MYB44*-mediated stomatal resistance in order to identify downstream events mediated by CF.

## Materials and Methods

### Plant materials

The seeds of wild-type *Arabidopsis thaliana* (Col-0), a T-DNA knockout line of *MYB44* (*myb44*, Salk_008606C), and overexpression line of MYB44 (MYB44ox, F19506) (7) were grown using the hydroponic culture system described by Toda *et al.* (29) for 2 weeks in all experiments. Briefly, *Arabidopsis* seeds were soaked in 0.5 mL of distilled water in 1.5-mL Eppendorf tubes, kept at 4°C for 2 d, and then placed on a nylon mesh (50 holes per inch) (Filter-net, Sansyo, Tokyo, Japan) held in a plastic photo slide mount (FujiFilm, Tokyo, Japan) (18 seeds per mount). The mounts were floated on 1/5 strength MGRL nutrients (pH 5.6) (5) and grown at 22°C under ~60 μE m^−2^ s^−1^ and 16/8-h (light/dark) conditions. The MGRL solution was renewed once a week after the start of the culture.

### Culture of the fungus and bacterium

The PGPF *P. simplissimum* GP17-2 (GP17-2) isolated from the rhizosphere of zoysiagrass (*Z. tenuifolia*) (18, 19) was maintained in potato dextrose agar (PDA) medium at 4°C and as a barley grain inoculum (BGI) at −20°C. In order to recover GP17-2, the BGI was placed on PDA medium in a 9-cm Petri dish and grown at 25°C for 7 d. The culture was then used to prepare CF.

The bacterial pathogen *P. syringae* pv. *tomato* DC3000 (*Pst*) was maintained as a 40% (v/v) glycerol stock at −80°C. In order to recover the *Pst*, the glycerol stock was cultured on a King’s medium B (KB) plate with 5 μg mL^−1^ rifampicin at 25°C for 3 d. A single colony was selected and transferred to a 100-mL Erlenmeyer flask containing 50 mL of liquid King’s medium B with 5 μg mL^−1^ rifampicin, and the culture was shaken at 130 rpm at 25°C for 2 d and then used for inoculation.

### Preparation and treatment of CF

Twenty mycelial disks (8 mm in diameter) cut out from the colony margin on PDA were transferred to a 500-mL Erlenmeyer flask containing 200 mL of potato dextrose broth (PDB) and incubated without shaking at 25°C for 10 d in the dark. The culture broth was filtered through two layers of filter paper (Whatman qualitative filter paper No. 2, GE Healthcare Japan, Hino, Japan), and CF was then filter-sterilized using a 0.22-μm Millex-GV syringe filter unit (Merck Millipore, Darmstadt, Germany). The CF treatment was performed 1 d before the challenge inoculation by dipping the roots of 2-week-old plants into CF for 1 h. Control plants were treated with PDB or sterile distilled water (SDW) instead of CF.

### Inoculation with the pathogen and disease assessment

CF-treated and PDB-treated plants were placed at 100% relative humidity for 23 h. Cultured bacterial cells, as described above, were collected by centrifugation at 3,000 rpm for 10 min, washed twice, and re-suspended with 10 mM MgSO_4_ supplemented with 0.01% (v/v) Silwet L-77 (Nihon Unica, Tokyo, Japan) to a concentration with OD_600_ 0.07 (3.5×10^7^ cfu mL^−1^). The challenge inoculation was performed by foliar spraying with the *Pst* suspension. Inoculated plants were incubated at 22°C and 100% relative humidity in the dark for 2 d and then under light for 1 d. The shoots were harvested, weighed, surface-sterilized twice by dipping in 100% Et-OH for 1 s, and immediately rinsed with sterilized distilled water. The shoots were then homogenized with a pestle in 1.5-mL Eppendorf tubes containing 1 mL of SDW, and 100-μL aliquots of the appropriate dilutions were then spread onto KB plates containing 50 μg mL^−1^ rifampicin. After a 48-h incubation at 25°C, *Pst* colonies (colony-forming units, cfu) were counted and determined as cfu gram^−1^ fresh weight of tissue. At least 3–6 replicates per treatment were analyzed. One replicate used six plants.

### Measurement of the stomatal aperture

Two-week-old plants were treated with CF as described above. Similarly, plants were treated with 100 μM ABA, SDW, or PDB. Plants were inoculated 23 h after the treatments with *Pst* using the inoculum prepared, as described above, by shoot immersion and incubated at 22°C under light. Leaves were harvested before and 1 and 3 h after the inoculation, and the abaxial surface was immediately coated with collodion (Ekivan A, Taihei Medicine, Ibaraki, Japan) to take an impression of the stomata. The impression was observed under a light microscope (Olympus BX-70, Tokyo, Japan) at 400× magnification, and images of the stomata were acquired randomly from five leaves of five different plants. The widths of the stomata (*n*=60) were measured using Pix2000_Pro software (Inotech, Hiroshima, Japan). Experiments were performed in triplicate.

### Gene expression analysis

Two-week-old plants treated with CF and PDB by the same method used for the measurement of the stomatal aperture following the inoculation were harvested for RNA extraction at the indicated time points. Samples were collected in 1.5-mL Eppendorf tubes and ground in liquid N_2_ with a pestle. Total RNA was extracted using Sepasol^®^-RNA I Super G (Nacalai Tesque, Kyoto, Japan) following the manufacturer’s protocol. The concentrations of the extracted RNA were measured with a spectrophotometer (BioSpectrometer^®^ Basic; Eppendorf, Tokyo, Japan), and 500 ng of total RNA was used to synthesize first-strand cDNA by ReverTra Ace qPCR RT Master Mix with a gDNA Remover (TOYOBO, Osaka, Japan), following the manufacturer’s protocol. The reverse transcription products (10 μL) were diluted by one half with sterile water and used as templates for real-time quantitative PCR (qRT-PCR) performed using SYBR^®^ Premix Ex Taq™ II (Tli RNaseH Plus) (TaKaRa, Otsu, Japan). qRT-PCR reaction mixtures were prepared in a total volume of 10 μL containing 5 μL of 2×SYBR Premix, 0.4 μL of 10 μM each of the forward and reverse primers (0.4 μM final concentration), 0.8 μL of the cDNA template, and 3.4 μL of dH_2_O. Gene-specific primers for *MYB44* (AT5G67300) were forward 5′-TCTCCACCTGTTGTTACTGGGCTT-3′ and reverse 5′-TTGA CTCGTGGCTACGGTTTGACT-3′ (8) and for *Actin2* (AT3G18780) as an internal control were forward 5′-GGCAAGTCATCACGA TTGG-3′ and reverse 5′-CAGCTTCCATTCCCACAAAC-3′ (17). The reactions were performed with a Thermal Cycler Dice^®^ Real Time System II (TP900, TaKaRa) under the following conditions: an initial denaturation step of 95°C for 30 s, 40 cycles of the two-step thermal cycling profile of denaturation at 95°C for 5 s, and primer annealing and extension at 60°C for 60 s. In order to verify the specificity of the amplicon for each primer pair, the final dissociation step was performed at 95°C for 15 s, 60°C for 30 s, and 95°C for 15 s. The relative standard curve method was used for the quantification of mRNA expression. cDNA standard curves were prepared using the threshold cycles with five serial dilution series (diluting cDNA samples to 1/2, 1/4, 1/8, 1/16, and 1/32). At least three technical replicates were analyzed for each sample. Five plants per condition were used.

## Results

### Reduction in Pst resistance induced by CF in the *myb44* mutant

**Fig. 1:**
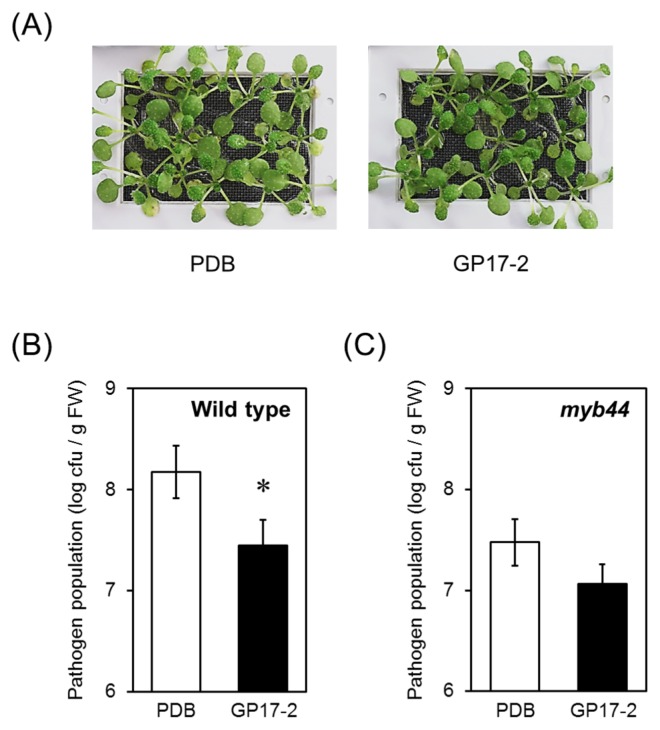
Systemic resistance induced by *Penicillium simplicissimum* GP17-2. Wild-type *Arabidopsis* plants (A, B) and *myb44* mutants (C) were grown for 2 weeks by a hydroponic culture in MGRL. Plants were treated with the culture filtrate from *Penicillium simplicissimum* GP17-2 or PDB as a control and then inoculated with *Pseudomonas syringae* pv. *tomato* DC3000 (*Pst*) followed by an incubation at 22°C in the dark for 2 d and then under light for 1 d. *Pst* colonies (colony forming units, cfu) were counted and determined as cfu gram^−1^ fresh weight. Plots show means ± SE of four independent experiments. ^*^, significant difference based on the Student’s *t*-test (*P*<0.05).

The *Pst* population and symptoms in wild-type plants were significantly smaller and weaker, respectively, with the CF treatment (GP17-2) than with the control (PDB) (Fig. 1A and B). In contrast, no significant difference was observed in the pathogen population in CF-treated *myb44* mutants (Fig. 1C).

### Stomatal reopening was restricted by CF and ABA

**Fig. 2:**
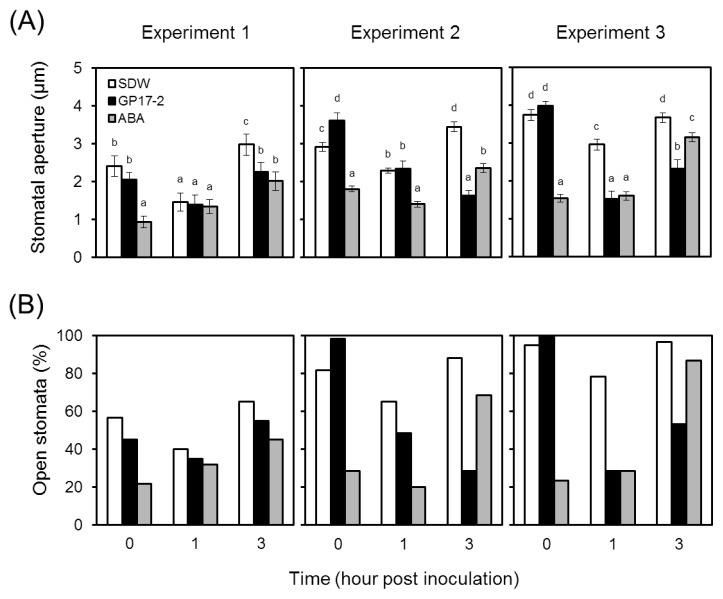
Stomatal closure by *Penicillium simplicissimum* GP17-2 or the ABA treatment during *Pseudomonas syringae* pv. *tomato* DC3000 infection. Two-week-old plants were treated with the culture filtrate from *Penicillium simplicissimum* GP17-2 (GP17-2, closed bar), 100 μM ABA (ABA, shaded bar), and, as a control, SDW (SDW, open bar). Stomatal apertures were measured before the inoculation (0 h) and 1 and 3 h after the inoculation with *Pseudomonas syringae* pv. *tomato* DC3000. Plots show (A) means ± SE and (B) the ratio of open stomata (*n*=60, consisting of five plants). Different letters show significant differences based on Tukey’s multiple comparison test (*P*<0.01). Results were confirmed by three independent experiments (Experiments 1–3).

As shown in Fig. 2, the stomatal aperture of control plants (SDW) was reduced from before the inoculation (0 h) to 1 h after the inoculation, and closed stomata reopened 3 h after the inoculation. In CF-treated plants (GP17-2), the stomatal aperture was reduced from 0 to 1 h, a similar time frame to that required in control plants, and then reopened at 3 h, and was significantly weaker than that in control plants (decrease in the aperture size, 0.72–1.81 μm; decrease in open stomata, 10%–60% that of control plants). In ABA-treated plants (ABA), the stomatal aperture was significantly reduced without the inoculation (0 h), and reopening at 3 h was also markedly weaker than that in control plants (decrease in the aperture size, 0.52–1.10 μm; decrease in open stomata, 10%–20%), which was similar to that observed with the CF treatment (GP17-2).

### Induction of *MYB44* expression during ISR

**Fig. 3:**
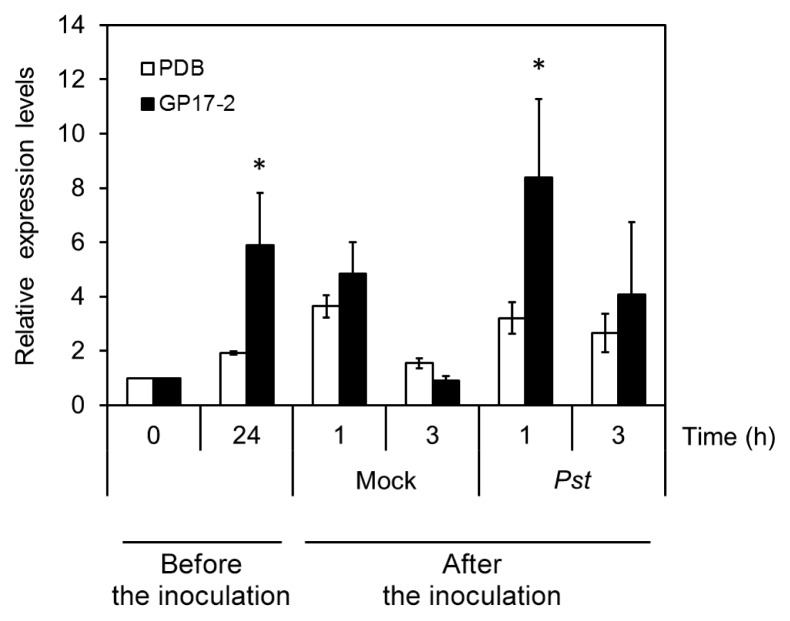
Expression analysis of *MYB44* in *Arabidopsis* during *Penicillium simplicissimum* GP17-2-elicited ISR against *Pseudomonas syringae* pv. *tomato* DC3000. Two-week-old plants were treated with the culture filtrate from *Penicillium simplicissimum* GP17-2 (GP17-2, closed bar) or PDB as a control (PDB, open bar). The plants were then inoculated with *Pseudomonas syringae* pv. *tomato* DC3000. The results of qRT-PCR showing *MYB44* expression levels normalized with *Actin2* at the indicated time are plotted. Error bars in the plot denote SE determined from at least three experiments. An asterisk denotes significant differences from the corresponding controls based on the Student’s *t*-test (*P*<0.05).

Fig. 3 shows that the treatment of *Arabidopsis* with CF increased the expression of *MYB44* 5.9-fold after 24 h, and the response observed was markedly stronger than that of the PDB control (Fig. 3, before inoculation). We then investigated the response of *MYB44* to the inoculation of *Pst* after the CF treatment. As shown in the figure, subsequent mock treatments resulted in a small and transient induction 1 h after the inoculation in CF-treated and control plants (Mock in the figure). The subsequent inoculation of *Pst* resulted in the selective enhancement of *MYB44* expression after 1 h in CF-treated plants, and this enhancement was reduced 3 h after the inoculation. In contrast, PDB control plants showed no enhancement by the inoculation.

### Requirement of *MYB44* for stomatal closure by the CF treatment

**Fig. 4:**
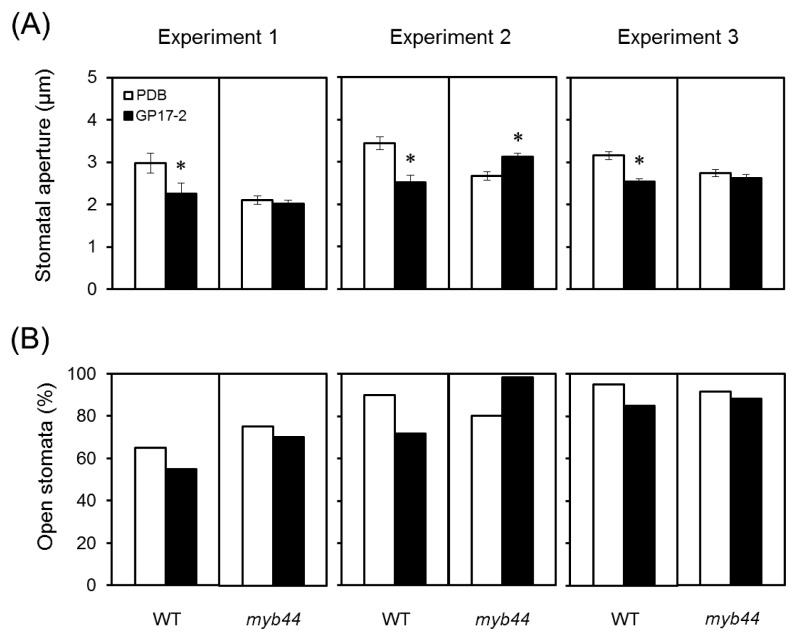
Involvement of MYB44 in stomatal closure by *Penicillium simplicissimum* GP17-2-elicited ISR. Two-week-old plants of *Arabidopsis* wild-type and *myb44* mutants were treated with the culture filtrate from *Penicillium simplicissimum* GP17-2 (GP17-2, closed bar) or PDB as a control (PDB, open bar). The stomatal aperture was measured 3 h after the inoculation with *Pseudomonas syringae* pv. *tomato* DC3000. Plots show (A) means ± SE and (B) the proportion of open stomata (*n*=60, consisting of five plants). Results were confirmed by three independent experiments (Experiments 1–3). An asterisk denotes significant differences from corresponding controls based on the Student’s *t*-test (*P*<0.05).

The stomatal aperture was measured 3 h after the inoculation in CF-treated wild-type and *myb44* mutant plants (Fig. 4). The stomatal aperture was smaller in CF-treated wild-type plants than in PDB-treated wild-type plants (decrease in the aperture size, 0.62–0.92 μm, decrease in open stomata, 10%–18%). In contrast, *myb44* plants showed no reduction in the stomatal aperture by the CF treatment.

**Fig. 5:**
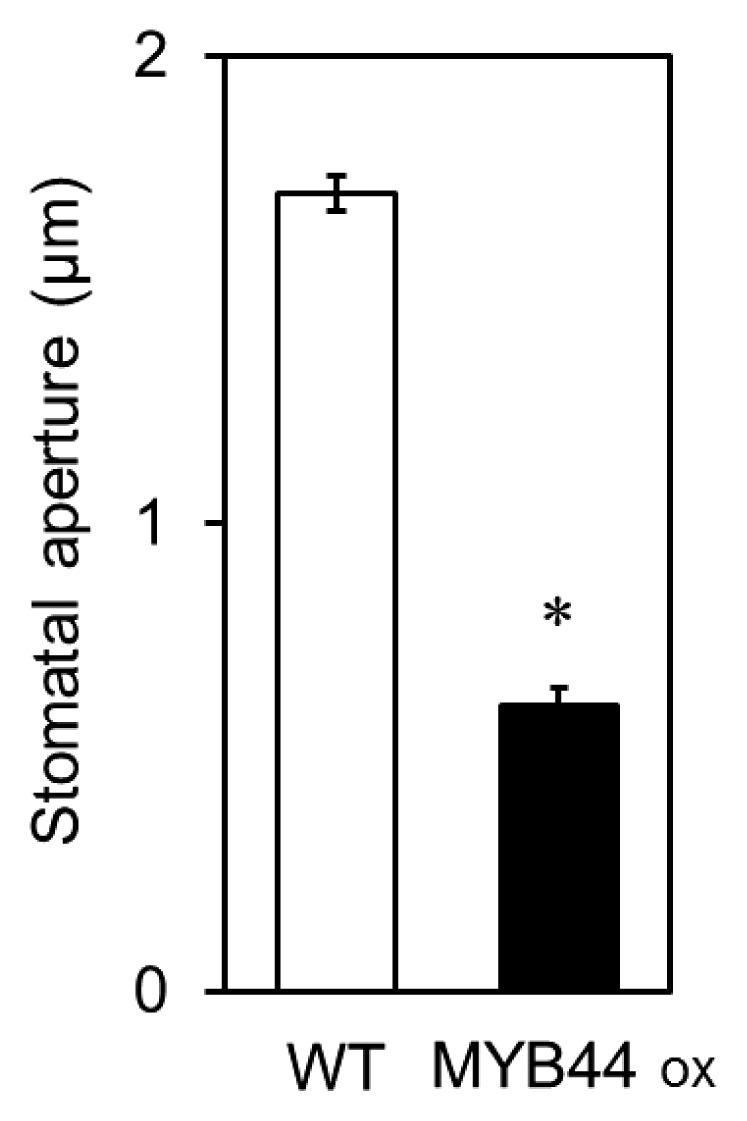
Smaller stomatal apertures in transgenic *Arabidopsis* plants overexpressing MYB44. Two-week old plants of wild-type and transgenic *Arabidopsis* overexpressing MYB44 were used to measure stomatal apertures under normal growth conditions. Plots show means ± SE (*n*=60, consisting of five plants) and are representative of two independent experiments. An asterisk means a significant difference from the corresponding controls based on the Student’s *t*-test (*P*<0.05).

The stomatal aperture was smaller in MYB44 ox plants than in wild-type plants (decrease in the aperture size, 1.1 μm) without elicitation or pathogen recognition (Fig. 5).

## Discussion

In our previous study, CF treatment-elicited ISR against *Pst* was identified in *Arabidopsis* (6). The restriction of stomatal reopening by *Pst* is an essential part of innate immunity against plant pathogenic bacteria (2, 3, 14, 15, 20–22, 28, 31–33). We found that CF-treated plants showed induced stomatal resistance against *Pst* (Fig. 2). The elicitor activity of CF has been detected in a dialysis fraction (>MW 12,000) (13). Since this activity did not disappear following a treatment with protease, autoclaving, or the removal of lipids, we concluded that the main elicitor compound is polysaccharide(s) (13). The same elicitor may trigger stomatal resistance against *Pst*. Since the primed state of stomatal resistance was sustained for 1 d, we hypothesized that CF-mediated stomatal resistance is achieved through transcriptional regulation. We focused on the transcription factor, *MYB44* (AT5G67300) because it is a stomata-specific enhancer of ABA signaling for stomatal closure (9) and has been identified as an elicitor-responsive gene (16, 25). These features led us to the hypothesis of the involvement of *MYB44* in stomatal regulation by CF. We investigated the hypothesis that *MYB44* mediates GP17-2- elicited ISR by stomatal closure. The expression profiles (Fig. 3) and phenotypes of *MYB44* mutants (Fig. 1 and 4) supported our hypothesis, demonstrating that the gene activation of *MYB44* for stomatal regulation is an essential part of ISR. One advantage of stomatal regulation by gene activation over rapid regulation at the protein level is the longer persistence of the regulated state of stomata, which supports the long memory of the priming effects of CF. *myb44* mutant plants did not fail to close their stomata 1 h after the inoculation (data not shown), indicating that *MYB44* is not essential for stomatal closure as innate immunity, but is important for supporting long-term stomatal resistance.

Although ISR by GP17-2 involves stomatal regulation, as discussed above, we do not exclude the additional possibility of the promotion of ISR, which involves the modulation of phytohormone effects by the pathogen infection. *Pst* invades plants by suppressing their SA-mediated defense responses through the activation of the JA pathway (34). In the present study, we identified the involvement of *MYB44* in the process of GP17-2-elicited ISR (Fig. 1, 3, and 4). As reported previously, *MYB44* enhances ABA signaling (9), and the ABA signal is known to be antagonistic to pathogen-associated JA signaling (1). Thus, the role of *MYB44* in ISR may include antagonistic activity against JA signaling. *MYB44* has also been shown to modulate SA signaling (26). The overexpression of *MYB44* activates *WRKY70* by directly binding to its promoter, thereby enhancing SA-mediated defense responses (26). Thus, the gene activation of *MYB44* by CF may also activate SA-mediated defense responses. The relationship between GP17-2-elicited ISR and SA responses currently remains unknown.

Several studies have investigated the gene activation of *MYB44*, and showed that it is activated by environmental stresses (dehydration, low temperature, salinity, and wounding), pathogenic elicitors (flg22 and harpin protein [HrpN_Ea_]), and stress-associated hormones (methyl jasmonate and ABA) (8–10, 16, 25, 30). It has not yet been established whether an unidentified elicitor in CF directly activates *MYB44* or if gene activation is achieved via modulation by an unidentified phytohormone.

The treatment of plants with elicitors with the ability to induce stomatal closure, including flg22, a yeast elicitor, and fungal elicitor (chitosan), is accompanied by ROS accumulation, NO synthesis, and (Ca^2+^)_cyt_ oscillations (11, 12). These accompanying events are all common to ABA signaling. Thus, stomatal regulation by elicitors is suggested to share a common mechanism with ABA signaling. The results of the present study, which have revealed the involvement of *MYB44*, an ABA regulator, are consistent with this suggestion.

Stomatal closure is enhanced by root colonization by the rhizobacterium *Bacillus subtilis* FB17 (FB17) in order to restrict pathogen entry (14). ABA and SA pathways are considered to regulate the stomatal aperture in FB17-elicited ISR (14). This is also consistent with the finding of the involvement of *MYB44*, which activates ABA and WRKY70-mediated SA signals.

**Fig. 6:**
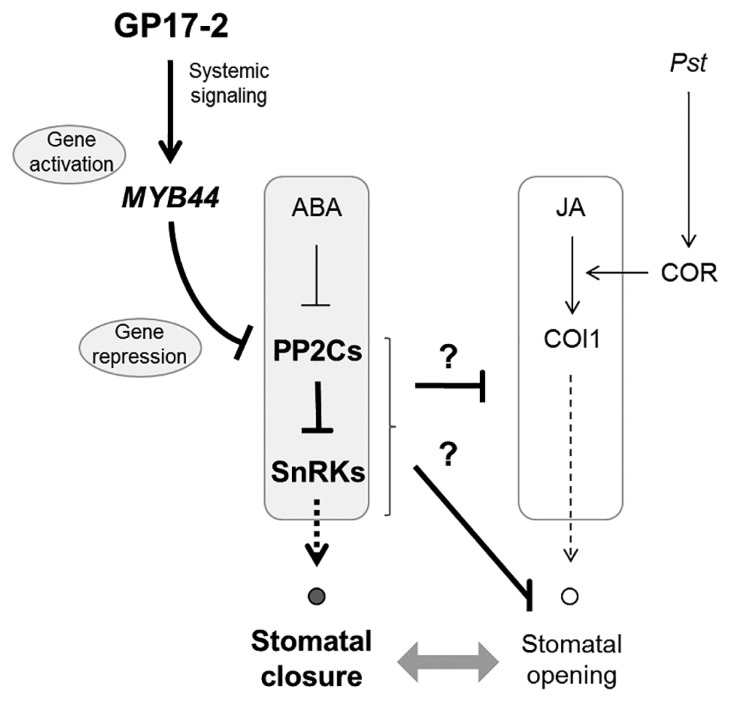
Model for effects of MYB44 in *Penicillium simplicissimum* GP17-2-elicited ISR. *Penicillium simplicissimum* GP17-2 (GP17-2) stimulates disease resistance in plants in part by stomatal closure (Fig. 1 and 2). Stomatal closure by GP17-2 is achieved by the enhancement of ABA signaling through the gene activation of *MYB44* (Fig. 3 and 4). Activated MYB44 represses PP2Cs, leading to the activation of ABA signaling (9). Coronatine (COR) produced by *Pseudomonas syringae* pv. *tomato* DC3000 (*Pst*) forces stomatal reopening by inducing COI1-mediated JA signaling in plants (20). SnRKs, Snf1-related protein kinases. Open and closed circles indicate ion channels regulating the stomatal aperture.

Fig. 6 shows a summary of our results and the reported function of MYB44. GP17-2 induces disease resistance in plants partly via stomatal closure (Fig. 1 and 2). The GP17-2- induced suppression of stomatal reopening is mediated by the activation of *MYB44* at the level of gene expression (Fig. 3 and 4). Activated *MYB44* represses protein phosphatase 2Cs (PP2Cs) (*ABI1*, *ABI2*, *AtPP2CA*, *HAB1*, and *HAB2*) by the transcriptional suppression of PP2C genes through a repressor domain in MYB44 (9, 23). This suppression of PP2Cs causes the activation of ABA signaling (9). Therefore, the stomatal aperture was smaller in MYB44 ox plants than in wild-type plants (Fig. 5), which is consistent with previous findings (9). We demonstrated that CF-enhanced *MYB44* expression did not result in the closure of the stomata prior to the inoculation (Fig. 2), whereas MYB44 ox plants exhibited stomatal closure (Fig. 5). These results suggest that *MYB44*-mediated stomatal closure is moderately regulated and will be enhanced by pathogen recognition. In contrast, COR produced by *Pst* has been shown to force stomatal reopening (20). Since the chemical structure of COR is similar to that of JA-Ile, COR has been suggested to activate JA signaling as an analog of JA-Ile (20). There are two possible mechanisms by which MYB44 suppresses stomatal reopening by COR. One is the antagonistic interaction of GP17-2-enhanced ABA signaling with pathogen-associated JA signaling (upper question mark in the figure). Another is the regulation of ion channels (open and closed circles in the figure) in guard cells by GP17-2-enhanced ABA signaling (lower question mark in figure).

In conclusion, our study on GP17-2-elicited ISR suggest that the involvement of *MYB44* in ISR and *MYB44*-mediated stomatal resistance is a common event in ISR.

## References

[1] Anderson J.P., Badruzsaufari E., Schenk P.M., Manners J.M., Desmond O.J., Ehlert C., Maclean D.J., Ebert P.R., Kazan K. (2004). Antagonistic interaction between abscisic acid and jasmonate-ethylene signaling pathways modulates defense gene expression and disease resistance in Arabidopsis. Plant Cell.

[2] Desclos-Theveniau M., Arnaud D., Huang T.Y., Lin G.J., Chen W.Y., Lin Y.C., Zimmerli L. (2012). The Arabidopsis lectin receptor kinase LecRK-V.5 represses stomatal immunity induced by *Pseudomonas syringae* pv. *tomato* DC3000. PLoS Pathog.

[3] Du M., Zhai Q., Deng L. (2014). Closely related NAC transcription factors of tomato differentially regulate stomatal closure and reopening during pathogen attack. Plant Cell.

[4] Elsharkawy M.M., Shimizu M., Takahashi H., Hyakumachi M. (2012). Induction of systemic resistance against *Cucumber mosaic virus* by *Penicillium simplicissimum* GP17-2 in *Arabidopsis* and tobacco. Plant Pathol.

[5] Fujiwara T., Yokota-Hirai M., Chino M., Komeda Y., Naito S. (1992). Effects of sulfur nutrition on expression of the soybean seed storage protein genes in transgenic petunia. Plant Physiol.

[6] Hossain M.M., Sultana F., Kubota M., Koyama H., Hyakumachi M. (2007). The plant growth-promoting fungus *Penicillium simplicissimum* GP17-2 induces resistance in *Arabidopsis thaliana* by activation of multiple defense signals. Plant Cell Physiol.

[7] Ichikawa T., Nakazawa M., Kawashima M. (2006). The FOX hunting system: an alternative gain-of-function gene hunting technique. Plant J.

[8] Jaradat M.R., Feurtado J.A., Huang D., Lu Y., Cutler A.J. (2013). Multiple roles of the transcription factor AtMYBR1/AtMYB44 in ABA signaling, stress responses, and leaf senescence. BMC Plant Biol.

[9] Jung C., Seo J.S., Han S.W., Koo Y.J., Kim C.H., Song S.I., Nahm B.H., Choi Y.D., Cheong J.J. (2008). Overexpression of *AtMYB44* enhances stomatal closure to confer abiotic stress tolerance in transgenic Arabidopsis. Plant Physiol.

[10] Jung C., Shim J.S., Seo J.S., Lee H.Y., Kim C.H., Choi Y.D., Cheong J.J. (2010). Non-specific phytohormonal induction of AtMYB44 and suppression of jasmonate-responsive gene activation in *Arabidopsis thaliana*. Mol Cells.

[11] Khokon M.A., Hossain M.A., Munemasa S., Uraji M., Nakamura Y., Mori I.C., Murata Y. (2010). Yeast elicitor-induced stomatal closure and peroxidase-mediated ROS production in Arabidopsis. Plant Cell Physiol.

[12] Klusener B., Young J.J., Murata Y., Allen G.J., Mori I.C., Hugouvieux V., Schroeder J.I. (2002). Convergence of calcium signaling pathways of pathogenic elicitors and abscisic acid in Arabidopsis guard cells. Plant Physiol.

[13] Koike N., Hyakumachi M., Kageyama K., Tsuyumu S., Doke N. (2001). Induction of systemic resistance in cucumber against several diseases by plant growth-promoting fungi: lignification and superoxide generation. Eur J Plant Pathol.

[14] Kumar A.S., Lakshmanan V., Caplan J.L., Powell D., Czymmek K.J., Levia D.F., Bais H.P. (2012). Rhizobacteria *Bacillus subtilis* restricts foliar pathogen entry through stomata. Plant J.

[15] Liu J., Elmore J.M., Fuglsang A.T., Palmgren M.G., Staskawicz B.J., Coaker G. (2009). RIN4 functions with plasma membrane H^+^-ATPases to regulate stomatal apertures during pathogen attack. PLoS Biol.

[16] Liu R., Chen L., Jia Z., Lü B., Shi H., Shao W., Dong H. (2011). Transcription factor AtMYB44 regulates induced expression of the *ETHYLENE INSENSITIVE2* gene in *Arabidopsis* responding to a harpin protein. Mol Plant Microbe Interact.

[17] Maruta T., Yonemitsu M., Yabuta Y., Tamoi M., Ishikawa T., Shigeoka S. (2008). *Arabidopsis* phosphomannose isomerase 1, but not phosphomannose isomerase 2, is essential for ascorbic acid biosynthesis. J Biol Chem.

[18] Meera M.S., Shivanna M.B., Kageyama K., Hyakumachi M. (1994). Plant growth promoting fungi from zoysiagrass rhizosphere as potential inducers of systemic resistance in cucumbers. Phytopathology.

[19] Meera M.S., Shivanna M.B., Kageyama K., Hyakumachi M. (1995). Persistence of induced systemic resistance in cucumber in relation to root colonization by plant growth promoting fungal isolates. Crop Prot.

[20] Melotto M., Underwood W., Koczan J., Nomura K., He S.Y. (2006). Plant stomata function in innate immunity against bacterial invasion. Cell.

[21] Melotto M., Underwood W., He S.Y. (2008). Role of stomata in plant innate immunity and foliar bacterial diseases. Annu Rev Phytopathol.

[22] Montillet J.L., Leonhardt N., Mondy S. (2013). An abscisic acid-independent oxylipin pathway controls stomatal closure and immune defense in *Arabidopsis*. PLoS Biol.

[23] Persak H., Pitzschke A. (2014). Dominant repression by Arabidopsis transcription factor MYB44 causes oxidative damage and hypersensitivity to abiotic stress. Int J Mol Sci.

[24] Pieterse C.M., Van der Does D., Zamioudis C., Leon-Reyes A., Van Wees S.C. (2012). Hormonal modulation of plant immunity. Annu Rev Cell Dev Biol.

[25] Pitzschke A., Djamei A., Teige M., Hirt H. (2009). VIP1 response elements mediate mitogen-activated protein kinase 3-induced stress gene expression. Proc Natl Acad Sci USA.

[26] Shim J.S., Jung C., Lee S., Min K., Lee Y.W., Choi Y., Lee J.S., Song J.T., Kim J.K., Choi Y.D. (2013). *AtMYB44* regulates *WRKY70* expression and modulates antagonistic interaction between salicylic acid and jasmonic acid signaling. Plant J.

[27] Shimizu K., Hossain M.M., Kato K., Kubota M., Hyakumachi M. (2013). Induction of defense responses in cucumber plants by using the cell-free filtrate of the plant growth-promoting fungus *Penicillium simplicissimum* GP17-2. J Oleo Sci.

[28] Singh P., Kuo Y.C., Mishra S. (2012). The lectin receptor kinase-VI.2 is required for priming and positively regulates Arabidopsis pattern-triggered immunity. Plant Cell.

[29] Toda T., Koyama H., Hara T. (1999). A simple hydroponic culture method for the development of a highly viable root system in *Arabidopsis thaliana*. Biosci Biotech Bioch.

[30] Wang Z., Cao G., Wang X., Miao J., Liu X., Chen Z., Qu L.J., Gu H. (2008). Identification and characterization of COI1-dependent transcription factor genes involved in JA-mediated response to wounding in *Arabidopsis* plants. Plant Cell Rep.

[31] Zeng W., He S.Y. (2010). A prominent role of the flagellin receptor FLAGELLIN-SENSING2 in mediating stomatal response to *Pseudomonas syringae* pv. *tomato* DC3000 in Arabidopsis. Plant Physiol.

[32] Zeng W., Brutus A., Kremer J.M., Withers J.C., Gao X., Jones A.D., He S.Y. (2011). A genetic screen reveals Arabidopsis stomatal and/or apoplastic defenses against *Pseudomonas syringae* pv. *tomato* DC3000. PLoS Pathog.

[33] Zhang W., He S.Y., Assmann S.M. (2008). The plant innate immunity response in stomatal guard cells invokes G-protein-dependent ion channel regulation. Plant J.

[34] Zheng X.Y., Spivey N.W., Zeng W., Liu P.P., Fu Z.Q., Klessig D.F., He S.Y., Dong X. (2012). Coronatine promotes *Pseudomonas syringae* virulence in plants by activating a signaling cascade that inhibits salicylic acid accumulation. Cell Host Microbe.

